# Usefulness of the delta neutrophil index in predicting surgery in patients with foot and ankle infection

**DOI:** 10.1371/journal.pone.0272574

**Published:** 2022-08-04

**Authors:** Ji eun Shin, Kyung Deok Seo, Hyun Jae Cha, Jong Wook Lee, Youn Moo Heo, Kwang Kyoun Kim, Tae Gyun Kim, Chan Kang, Gi Soo Lee, Jae Hwang Song

**Affiliations:** 1 Department of Biomedical Informatics, College of Medicine, Konyang University, Daejeon, Republic of Korea; 2 Department of Orthopedic Surgery, Konyang University Hospital, Daejeon, Republic of Korea; 3 Department of Laboratory Medicine, Konyang University Hospital, Daejeon, Republic of Korea; 4 Department of Orthopedic Surgery, Chungnam National University Hospital, Daejeon, Republic of Korea; University of Arizona Arizona Health Sciences Center: The University of Arizona Health Sciences, UNITED STATES

## Abstract

**Background:**

In foot and ankle infections, cases with apparent soft-tissue necrosis or purulent fluid collections definitely require surgical treatments. However, clinicians often have difficulty in determining whether to perform surgery in ambiguous cases without these findings. This study aimed to investigate the impact of the delta neutrophil index as a predictor of surgical treatment in patients with foot and ankle infections.

**Methods:**

In total, 66 patients diagnosed with foot and ankle infections who underwent the delta neutrophil index test were retrospectively investigated. Medical records, including data on diabetes mellitus status, delta neutrophil index values, white blood cell count, polymorphonuclear leukocyte count, erythrocyte sedimentation rate, and C-reactive protein level, were retrospectively investigated. Logistic regression models were analyzed for the correlation between biomarkers, such as the delta neutrophil index and surgical treatment. The area under the curve was investigated to evaluate the cut-off value of the logistic model in predicting surgery.

**Results:**

The relationship between the delta neutrophil index and surgical treatment was analyzed. The delta neutrophil index, adjusted for diabetes mellitus, was the best predictor of future surgical intervention. Based on the Youden index, the cutoff point (the equation’s adjusted by diabetes mellitus) for the prediction of surgical treatment was defined as a probability of 0.3, with sensitivity and specificity of 82.4% and 77.6%, respectively.

**Conclusions:**

Based on the present study, the delta neutrophil index can help clinicians decide the appropriate surgical treatment for foot and ankle infections at the right time.

## Introduction

Foot and ankle infections are common causes of morbidity, disability, and mortality [1, 2]. Foot and ankle infections pose a difficult and challenging treatment dilemma since patients often have poor vascular status and severe wound complications, such as in patients with diabetes mellitus and peripheral arterial occlusive disease [2]. Although cases with apparent soft-tissue necrosis or purulent fluid collections definitely require surgical treatments, including irrigation, debridement, or amputation [2], clinicians often have difficulty in determining whether to perform surgery in ambiguous cases without these findings.

To evaluate the severity of infection, history taking, physical examination, and radiographic evaluation are important. During physical examination, inspection should be performed to detect signs of infection, including tenderness, heating sense, erythema, swelling, blisters, and drainage [2]. For the radiographic evaluation, simple radiographs and magnetic resonance imaging studies can be used to obtain useful information regarding infective findings of the bone and soft tissue structure [2]. However, some limitations of the aforementioned tests remain due to their limited sensitivity and specificity [3].

Laboratory markers are another essential diagnostic tool that can quantitatively predict the severity and prognosis of infections. Laboratory markers, including white blood cell (WBC) count, polymorphonuclear leukocyte (PMN) count, erythrocyte sedimentation rate (ESR), and C-reactive protein(CRP) level, have been used for the diagnostic investigation of foot and ankle infections. However, these laboratory parameters cannot appropriately reflect the severity of foot and ankle inflammation; hence, it is difficult to predict necessity for operation in the patients [3–6]. Therefore, a novel serum biomarker that determines the need for surgery is required.

During infection, immature granulocytes (IGs) reveal the increased production of granulocytes [7], and it has been demonstrated that increased IGs is a useful biomarker for predicting infection [8]. However, manual counting of IGs needs considerable effort and time. The Delta neutrophil index (DNI) is a new, innovative parameter of the circulating fraction of IGs, and is automatically estimated using a cell analyzer without manual counting [9]. Recently, several studies reported that DNI significantly correlates with IGs, and proved the usefulness of DNI as a predictor of infection [4]. An increased DNI was related not only with severity of infection, but also with bacteremia, disseminated intravascular coagulation, and mortality in sepsis [4, 10–12].

Therefore, given the strong association with the severity of other inflammatory diseases, we hypothesized that the DNI would predict patients with foot and ankle infections in need of surgery. To the best of our knowledge, no previous studies have evaluated the correlation between DNI and foot and ankle infections. Hence, the present study aimed to investigate the usefulness of the DNI as a predictor of surgical treatment in patients with foot and ankle infections.

## Materials and methods

### Participants and subgroup analysis

**Fig 1:**
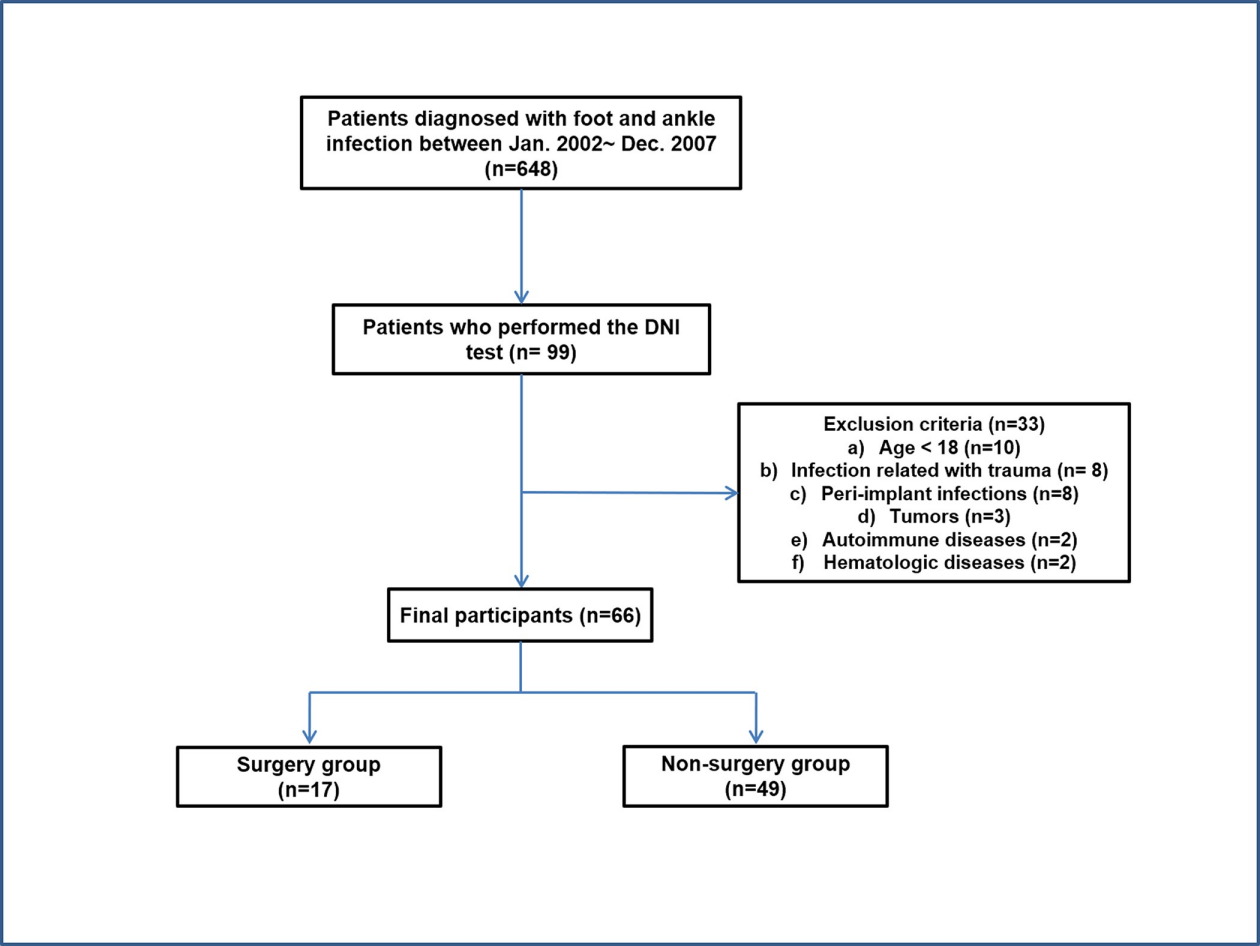
Flow diagram of participant eligibility.

The institutional review board (IRB) of Konyang University Hospital (KYUH 2020–06–025) approved this study. Following the guidelines for the diagnosis of skin and soft tissue infections by the infectious diseases society of America [13] and referring the most commonly used diagnostic terms associated with foot and ankle infections in our hospital, patients of our orthopedic department with the word “cellulitis; erysipelas; necrotizing fasciitis; furuncle; carbuncle; abscess; diabetic foot infection; septic arthritis; osteomyelitis” in discharge codes registered in computerized hospital records were initially considered as patients with foot and ankle infections. This study enrolled 99 of 648 patients with foot and ankle infections diagnosed at our hospital between 2002 and 2007 who underwent the DNI exam (Fig 1). We excluded patients aged <18 years, those with infection related to trauma (i.e., sprain or fracture), peri-implant infection, benign or malignant tumors, autoimmune diseases, and hematologic disorders. Finally, a cohort of 66 patients with foot and ankle infections were enrolled in the present study.

Data were collected retrospectively by reviewing medical records by two orthopedic surgeons who were blinded to the study. The following data were extracted from medical records: sex, age, admission day, diabetes mellitus (DM) status, inflammatory markers (measured on admission date, including DNI, WBC, PMN, ESR, and CRP), and other laboratory data (measured on admission date, including hemoglobin, hematocrit, platelet, prothrombin time, activated partial thromboplastin time, glucose, blood urea nitrogen, creatinine, total protein, albumin, sodium, potassium, and chloride).

To determine the treatment methods, signs of infection including erythema, swelling, blisters, heating sensation, pain, tenderness, drainage, as well as laboratory data, were thoroughly investigated by orthopedic surgeons who had more than 5 years of clinical experience. Initially, all patients underwent medical treatment with empirical intravenous (IV) antibiotics, such as Ampicillin-sulbactam [14] or cefazolin [15], regardless of surgical or non-surgical group. IV antibiotic treatment was maintained until the infection signs and laboratory outcomes were improved. Patients with extensive bone involvement, apparent purulent discharges, or soft tissue gangrene promptly underwent surgical treatments, including irrigation, debridement, or amputation. In ambiguous cases, the final decision regarding surgical intervention was made by the surgeon when abnormal laboratory outcomes and clinical signs persisted, despite medical treatment.

For statistical analysis, the participants were divided into two groups: the surgery and non-surgery groups. The aforementioned data obtained by reviewing the electronic medical records were compared between the two groups.

### Delta neutrophil index measurement

DNI is measured by the difference between leukocyte subfractions estimated using a nuclear lobularity assay and a cytochemical myeloperoxidase (MPO) stain. The DNI values were calculated by a hematology analyzer (ADIVA 120, Siemens, Inc.) by performing complete blood count (CBC) without additional cost or time, and the DNI values were calculated automatically and reported with the CBC results under prescription.

### Statistical analysis

Statistical and graphical analyses were performed using SPSS version 28.0 (IBM Corp., Armonk, NY, USA). Continuous data were compared between the two groups using Student’s t-test (parametric data) or the Mann-Whitney U test (nonparametric data). The Chi-squared or Fisher’s exact tests were used for the analysis of categorical data. Logistic regression analysis was used to calculate the predictive probability of each biomarker and combined biomarkers. To evaluate the cut-off value for predicting surgery, the receiver operating characteristic (ROC) curves and the area under the curve (AUC) were investigated. Each cut-off value was selected to maximize the sum of sensitivity and specificity. Statistical significance was set at p < 0.05 in all analyses.

## Results

Participants were divided into the surgery (n = 17) (Table 1) and non-surgery groups (n = 49). None of the patients included in the non-surgery group underwent surgery within 1 year follow up, and most of them needed intravenous antibiotic treatment for about 2–3 weeks.

**Table 1:** Baseline characteristics of the surgery group in the present study.

Patient number	Sex	Age	Diabetes mellitus	Diagnosis	Operation
1	M	74	+	Diabetic foot ulcer	Incision and drainage
2	F	91	+	Osteomyelitis	Amputation, 1^st^ toe
3	F	73	+	Osteomyelitis	Amputation, 1^st^ toe
4	M	87	+	Peripheral arterial occlusive disease	Disarticulation, 2^nd^ MTPJ
5	M	71	+	Osteomyelitis	Disarticulation, 4^th^ MTPJ
6	F	91	+	Peripheral arterial occlusive disease	Below knee amputation
7	M	76	+	Osteomyelitis	Disarticulation, 5^th^ MTPJ
8	M	84	+	Diabetic foot ulcer.	Incision and drainage
9	M	71	+	Peripheral arterial occlusive disease	Amputation, 4^th^ toe
10	M	67	+	Osteomyelitis	Disarticulation, 3^rd^ MTPJ
11	F	96	-	Osteomyelitis	Disarticulation, 5^th^ MTPJ
12	F	77	+	Diabetic foot ulcer	Incision and drainage
13	F	59	+	Diabetic foot ulcer	Incision and drainage
14	M	86	+	Peripheral arterial occlusive disease	Amputation, Lisfranc joint
15	M	45	+	Osteomyelitis	Disarticulation, 5^th^ MTPJ
16	M	50	-	Cellulitis, ankle	Incision and drainage
17	M	34	-	Cellulitis, foot	Incision and drainage

M, male; F, female; MTPJ, metatarsophalangeal joint.

The mean age, admission day, and rate of DM in the surgery group were significantly higher than those in the non-surgery group (p  < .001, respectively) (Table 2). In terms of inflammatory markers, DNI was the only marker that showed a significant difference between the two groups (p = .045). The mean of glucose (p < .001), blood urea nitrogen (p = .019), and potassium (p = .042) in the surgery group were significantly higher, and the mean of hemoglobin (p < .001), hematocrit (p  < .001), total protein (p = .035), and albumin levels (p < .001) were significantly lower than those in the non-surgery group.

**Table 2:** Baseline characteristics of participants with foot and ankle infection^a^.

Variables	Total	Surgery group	Non-surgery group	p value
	(n = 66)	(n = 17; 25.8%)	(n = 49; 74.2%)	
Demographic characteristics				
Sex				.528
Male	48 (72.7%)	11 (64.7%)	37 (75.5%)	
Female	18 (27.3%)	6 (35.3%)	12 (24.5%)	
Age (y)	54.7 (18–96)	72.5 (34–96)	48.6 (18–92)	< .001
Admission day (d)	21.5 (2–131)	42.6 (12–131)	14.2 (2–67)	< .001
Diabetes Mellitus	29 (43.9%)	14 (82.4%)	15 (30.6%)	< .001
Inflammatory markers				
DNI (%)	2.0 (0–8.4)	2.7(0–8.4)	1.8 (0–4.3)	.045
WBC (cells/μL)	12535.5 (4870–30710)	12147.7 (5160–30710)	12670 (4870–64700)	.220
PMN (%)	71.9 (46.7–87.9)	73.7 (52.4–87.9)	71.3 (46.7–86.1)	.153
ESR (mm/h)	34.0 (2–120)	36.5 (2–65)	33.2 (1–120)	.180
CRP (mg/dL)	6.9 (0.3–20.0)	6.4 (0.3–20.0)	7.1 (0.3–20.0)	.143
Other laboratory data				
Hb (g/dL)	13.0 (9.2–16.4)	11.5 (9.2–14.3)	13.5 (9.9–16.4)	< .001
Hct (%)	37.2 (28.6–49.9)	33.4 (28.6–40.1)	38.5 (28.6–49.9)	< .001
Plt (x1000/μL)	278.9 (128–586)	286.7 (128–586)	276.1 (134–536)	.686
PT (s)	13.7 (11.6–17.3)	13.7 (11.9–17.3)	13.6 (11.6–15.7)	.481
aPTT (s)	37.8 (2.6–63.9)	37.9 (25.6–63.9)	37.8 (2.6–56.0)	.090
Glucose (mg/dL)	171.0 (70–517)	267.8 (70–517)	137.4 (79–266)	< .001
BUN (mg/dL)	16.0 (6.6–52.4)	19.0 (6.6–52.4)	14.9 (7.0–50.0)	.019
Cr (mg/dL)	1.1 (0.6–4.6)	1.1 (0.7–3.3)	1.1 (0.6–4.6)	.496
Total protein (g/dL)	6.9 (5.5–8.1)	6.6 (5.5–7.9)	7.1 (5.9–8.1)	.035
Albumin (g/dL)	3.8 (2.5–4.7)	3.5 (2.5–4.3)	4.0 (2.5–4.7)	< .001
Na (mmol/L)	137.2 (128.0–144.0)	135.5 (128.0–143.0)	137.8 (132.0–144.0)	.067
K (mmol/L)	4.2 (3.4–5.9)	4.4 (3.9–5.7)	4.2 (3.4–5.9)	.042
Cl (mmol/L)	102.6 (91.6–109.0)	102.1 (91.6–108.0)	102.8 (94.2–109.0)	.493

^a^Values are presented as the mean and range (min–max). Boldface indicates a statistically significant difference between the two groups (p < .05).

DNI, delta neutrophil index; WBC, white blood cell; PMN, polymorphonuclear leukocyte; ESR, erythrocyte sedimentation rate; CRP, C-reactive protein; Hb, hemoglobin; Hct, hematocrit; Plt, platelet; PT, prothrombin time; aPTT, activated partial thromboplastin time; BUN, blood urea nitrogen; Cr, creatinine; Na, sodium; K, potassium; Cl, chloride.

Based on the logistic regression analysis for inflammatory biomarkers, DNI was the only significant predictor of surgical intervention (Table 3); as DNI increased, the odds ratio (OR) was 1.512-fold higher (95% confidence interval [CI], 1.013–2.257, p = 0.043).

**Table 3:** Predictors of surgical intervention for foot and ankle infection as determined by univariate logistic regression analysis.

Variables	OR	95% CI	p value
DNI (%)	1.512	1.013–2.257	.043
WBC (cells/μL)	1.000	1.000–1.000	.607
PMN (%)	1.042	0.971–1.118	.255
ESR (mm/h)	1.010	0.981–1.039	.511
CRP (mg/dL)	0.931	0.824–1.051	.245

DNI, delta neutrophil index; WBC, white blood cell; PMN, polymorphonuclear leukocyte; ESR, erythrocyte sedimentation rate; CRP, C-reactive protein; OR, odds ratio; CI, confidence interval.

**Fig 2:**
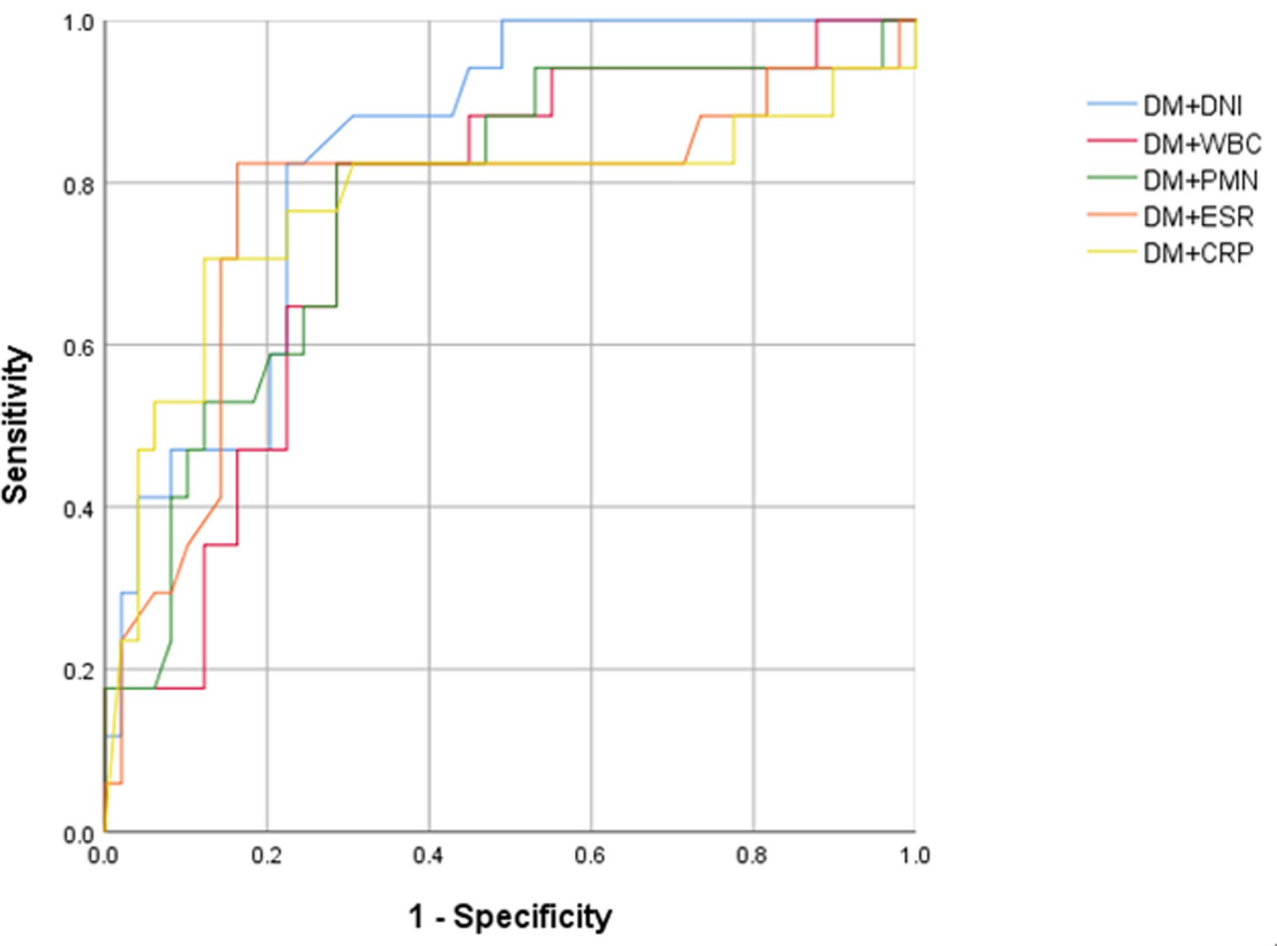
ROC curves of inflammatory biomarkers combined with DM for predicting surgery in patients with foot and ankle infection (DM, diabetes mellitus; DNI, delta neutrophil index; WBC, white blood cell; PMN, polymorphonuclear leukocyte; ESR, erythrocyte sedimentation rate; CRP, C-reactive protein).

Given the strong association between DM and surgery for the treatment of foot and ankle infections, we analyzed inflammatory markers adjusted by DM modalities (Table 4). The ROC curve and AUC were investigated using the p-value estimated by logistic regression analysis of combined DM modalities [16]. The cut-off ranges (cutofflogitP) of logitP (fitted equations) and corresponding incidence (Pcutoff) were estimated using the maximum Youden index [16]. Based on the logitP, the Pcutoff value of DNI was 0.3, and its sensitivity and specificity were 82.4% and 77.6%, respectively, with an AUC of 0.839 (95% CI, 0.742–0.937, p  < .001). As a result, the combination of DM and DNI was the most powerful method for predicting surgical interventions (Fig 2).

**Table 4:** Combined DM modalities of the inflammatory markers.

Model	Cut-offlogit *P*	P_cutoff_	_logit_ P	AUC (95% CI)	Sensitivity, %	Specificity, %	p value
DM+DNI	-0.849	0.300	-3.55+2.59DM+0.44DNI	0.839 (0.742–0.937)	82.4	77.6	< .001
DM+WBC	-0.13	0.468	-2.56+2.38DM+0.00WBC	0.759 (0.630–0.887)	82.4	71.4	.002
DM+PMN	-0.27	0.433	-3.09+2.33DM+0.01PMN	0.777 (0.645–0.909)	82.4	71.4	.001
DM+ESR	-0.254	0.437	-2.11+2.60DM-0.01ESR	0.772 (0.620–0.923)	82.4	83.7	.001
DM+CRP	-0.106	0.473	-2.26+2.39DM-0.03CRP	0.775 (0.616–0.934)	70.6	87.8	.001

DM, diabetes mellitus; DNI, delta neutrophil index; WBC, white blood cell; PMN, polymorphonuclear leukocyte; ESR, erythrocyte sedimentation rate; CRP, C-reactive protein; AUC, area under the curve; CI, confidence interval.

## Discussion

The findings of this study support our hypothesis that DNI could predict surgical treatment in patients with foot and ankle infections. The combination of DM and DNI revealed the highest predictive power for surgical treatment in these patients.

The foot and ankle have a distinctive risk of infection since they have important role in weight bearing and frequent exposure to trauma. Also their treatment is challenging since it is often affected by poor vascular supply or sensations related with diseases such as diabetes. Although many cases of foot and ankle infections can resolve with medical treatment, operative treatment is required in patients refractory to non-operative treatment. However, predicting the necessity of operation in patients with foot and ankle infections is challenging, since the clinical course varies depending on disease severity. In particular, determining surgery, such as amputation, can be a huge dilemma for surgeons and patients. However, a meaningless delay may aggravate morbidities such as gangrenous changes, or mortality. For this reason, it is important to timely determine the need of surgery in foot and ankle infections using objective diagnostic tools. To this end, researchers have sought novel markers that can be used to identify patients most likely to benefit from surgical treatment [17].

In the early period of sepsis and infection, immature neutrophils enter the circulation to compensate for the lack of active neutrophils, thereby causing a “leftshift” [18]. In this setting, the number of neutrophil bands, which indicate the amount of immature neutrophils, is increased [19]. Using IGs for evaluating infection has been recently suggested by several investigators [20]. However, counting granulocyte parameters is difficult, and methods for reliable quantification have not been established [4]. Furthermore, measuring IGs is labor-intensive and time-consuming.

On the contrary, the DNI is a measured value that reveals the ratio of IGs to the total neutrophil count [21]. The DNI is assessed by an automatic system through the nuclear lobularity and MPO channels [22]. By using an automatic cell analyzer, clinicians can easily obtain the DNI value which reports the index of the IGs fraction. The DNI has been reported to have a significant relationship with the severity of several infectious diseases [3]. Also, many previous studies have suggested the DNI as a useful predictor of surgical decisions [3]. Furthermore, no additional cost or time is necessary for obtaining the DNI [11]. Most importantly, the DNI has been reported to be a better predictor of infection and prognosis compared to traditional markers, including WBC count, ESR, and CRP [4–6].

The diagnosis of infection can be difficult if WBC values are in the normal or lower range because of leukopenic diseases, including tuberculosis and typhoid fever [4]. Instead, the DNI can diagnose and predict infections in patients with high accuracy, since the proportion of IGs is elevated even under the condition of normal WBC or absolute neutrophil count (ANC) [23].

ESR and CRP are commonly used biomarkers for the diagnosis and monitoring of infection. They can also provide accurate information related with inflammatory symptoms in orthopedic conditions [24]; however, there are some limitations associated with their use. Since an increase of ESR is induced by the rouleaux formation of red blood cells, ESR is rather insensitive to minor infection and its response to inflammation is quite slow [24]. Hence, ESR is currently not recommended as a screening test [25]. CRP is a preferred biomarker for acute inflammatory conditions since it shows more rapid kinetics and shorter half-life [26]. Therefore, it is useful for investigating response to treatment as well as diagnosis of infection. However, CRP levels can increase in several situations which cause tissue injury, including surgery, malignancies, and trauma [14]. In addition, CRP is not a specific parameter for infection-induced inflammation, as it can be increased in systemic autoimmune diseases, such as rheumatoid arthritis.

Procalcitonin has been used for the identification of bacterial infections [17] because of several advantages over other biomarkers, such as the wide biological range and short time of induction after bacterial infection. Thus procalcitonin has been widely used to guide the initiation and termination of antibiotics in various bacterial diseases [27]. In a systematic review and meta-analysis, the DNI’s pooled sensitivity and specificity as a predictive factor for infection were 0.67 (95% CI, 0.62–0.71) and 0.94 (95% CI, 0.94–0.95), respectively, with an AUC of 0.89 [4]; these results were comparable to those of CRP [28] and procalcitonin [29] as predictive factors for infection in previous studies. Since both CRP and procalcitonin levels elevate several hours after disease onset [30] while the DNI increases 12 h before the initiation of organ failure in patients with severe infection [31], the DNI can help diagnose and initiate treatment against infections faster [4]. Also, the DNI has much shorter life than procalcitonin, which is helpful during follow-up for therapeutic efficacy [4].

Among the inflammatory biomarkers, the DNI was the only marker that showed a significant difference between the surgery and non-surgery groups, and it was the only significant predictor of surgical intervention for foot and ankle infections. Among the baseline characteristics, DM and glucose levels differed significantly between the two groups. Given the strong association between DM and surgical intervention for the treatment of foot and ankle infections, we investigated the combined DM modalities of inflammatory markers [16]. A previous study suggested that combining ROC curve and logistic regression analyses is feasible for identifying several disease markers [16]. We found that the combination of DM and DNI exhibited the highest predictive power for operative treatment patients.

In previous studies of other diseases, the DNI was suggested as a useful biomarker that could predict surgical intervention [3, 32]. For example, Lee et al. [32] suggested that the initial DNI level can be a useful predictor for determining surgical intervention in patients with intestinal obstruction. The area under the ROC curve of the initial DNI (0.543) was higher than that of CRP (0.460) and WBC (0.449) in these patients. Similarly, Son et al. [3] reported that the DNI may be a good predictor for determining the necessity for operative treatment in chronic rhinosinusitis patients. Also in that setting, the area under the ROC curve of the initial DNI (0.782) was higher than that of WBC (0.571) and ESR (0.600). Hence, the combination of DM and DNI in the present study can also be suggested as a useful predictor of surgical treatment, considering the high AUC of the ROC curve (0.839).

To our knowledge, this is the first study to investigate the correlation between the DNI and foot and ankle infections. We compared DNI with several other inflammatory markers, including WBC, PMN, ESR, and CRP, which are the most commonly used laboratory tests for the diagnosis and monitoring of foot and ankle infections.

Our study has some limitations that should be acknowledged. First, the study was limited by its retrospective and single-center design, and its small sample size. Second, due to the nature of the retrospective cohort study design, potential confounders may exist. However, potential confounders such as comorbidity of peripheral arterial occlusive disease or chronic kidney disease were not identified in the present study. Further prospective studies with a larger number of patients that will include investigation of these confounders are therefore required. Third, clinical and radiological evaluations, which might have been an important factor in predicting surgery, were not included in this study. Fourth, the inclusion rate of the study was low (15%) since not all patients with foot and ankle infection underwent DNI test; this is because some doctors (professors or residents) of the orthopedic department did not prescribe the DNI code at that time. Finally, the study was performed from 2002 to 2007, because calculation of DNI was not available after that period due to a change of the auto-analyzer type in our hospital. However, many medical centers are still using the DNI for laboratory tests related to diagnosing and monitoring various infectious diseases.

## Conclusions

The DNI, adjusted for DM, was the best predictor of future surgical intervention in patients with foot and ankle infections. We suggest that the DNI can help clinicians determine the appropriate surgical treatment for foot and ankle infections at the right time. Further prospective studies with larger number of patients are required to support our data and minimize the limitations of this study.

## Supporting information

S1 Data (SAV)

## References

[1] Pitts SR, Niska W, Burt CW (2008) National Hospital Ambulatory Medical Care Survey: 2006 emergency department summary. National health statistics reports; no 7. 18958996

[2] Anakwenze OA, Milby AH, Gans I, Stern JJ, Levin SL, et al. (2012) Foot and ankle infections: diagnosis and management. JAAOS-Journal of the American Academy of Orthopaedic Surgeons 20: 684–693. doi: 10.5435/JAAOS-20-11-684 23118134

[3] Son S, An HG, Park JS, Kim SH, In SM, et al. (2021) Delta neutrophil index levels can be a good indicator to predict patients with chronic rhinosinusitis who need surgery. Ear, Nose & Throat Journal: 01455613211058491. doi: 10.1177/01455613211058491 34818928

[4] Park JH, Byeon HJ, Lee KH, Lee JW, Kronbichler A, et al. (2017) Delta neutrophil index (DNI) as a novel diagnostic and prognostic marker of infection: a systematic review and meta-analysis. Inflammation Research 66: 863–870. doi: 10.1007/s00011-017-1066-y 28646289

[5] Bermejo-Martín JF, Tamayo E, Ruiz G, Andaluz-Ojeda D, Herrán-Monge R, et al. (2014) Circulating neutrophil counts and mortality in septic shock. Critical Care 18: 1–4. doi: 10.1186/cc13728 24524810 PMC4057453

[6] Yoon N-B, Son C, Um S-J (2013) Role of the neutrophil-lymphocyte count ratio in the differential diagnosis between pulmonary tuberculosis and bacterial community-acquired pneumonia. Annals of laboratory medicine 33: 105–110. doi: 10.3343/alm.2013.33.2.105 23482854 PMC3589634

[7] Seebach JD, Morant R, Rüegg R, Seifert B, Fehr J (1997) The diagnostic value of the neutrophil left shift in predicting inflammatory and infectious disease. American journal of clinical pathology 107: 582–591. doi: 10.1093/ajcp/107.5.582 9128272

[8] Nigro KG, O’Riordan M, Molloy EJ, Walsh MC, Sandhaus LM (2005) Performance of an automated immature granulocyte count as a predictor of neonatal sepsis. American journal of clinical pathology 123: 618–624. doi: 10.1309/73H7-K7UB-W816-PBJJ 15743752

[9] Nahm CH, Choi JW, Lee J (2008) Delta neutrophil index in automated immature granulocyte counts for assessing disease severity of patients with sepsis. Annals of Clinical & Laboratory Science 38: 241–246. 18715852

[10] Kang HS, Cha YS, Park KH, Hwang SO (2017) Delta neutrophil index as a promising prognostic marker of emergent surgical intervention for acute diverticulitis in the emergency department. PloS one 12: e0187629. doi: 10.1371/journal.pone.0187629 29091955 PMC5665552

[11] Kim OH, Cha YS, Hwang SO, Jang JY, Choi EH, et al. (2016) The use of delta neutrophil index and myeloperoxidase index for predicting acute complicated appendicitis in children. PloS one 11: e0148799. doi: 10.1371/journal.pone.0148799 26859663 PMC4747520

[12] Kong T, Kim TH, Park YS, Chung SP, Lee HS, et al. (2017) Usefulness of the delta neutrophil index to predict 30-day mortality in patients with ST segment elevation myocardial infarction. Scientific reports 7: 1–11. doi: 10.1038/s41598-017-15878-5 29146994 PMC5691079

[13] Stevens DL, Bisno AL, Chambers HF, Dellinger EP, Goldstein EJ, et al. (2014) Practice guidelines for the diagnosis and management of skin and soft tissue infections: 2014 update by the Infectious Diseases Society of America. Clinical infectious diseases 59: e10–e52. doi: 10.1093/cid/ciu444 24973422

[14] Ablij HC, Meinders AE (2002) C-reactive protein: history and revival. European Journal of Internal Medicine 13: 412–422. doi: 10.1016/s0953-6205(02)00132-2 12384129

[15] Roberts AD, Simon GL. Diabetic foot infections: the role of microbiology and antibiotic treatment; 2012. Elsevier. pp. 75–81. 10.1053/j.semvascsurg.2012.04.010 22817856

[16] Yang Q, Zhang P, Wu R, Lu K, Zhou H (2018) Identifying the best marker combination in CEA, CA125, CY211, NSE, and SCC for lung cancer screening by combining ROC curve and logistic regression analyses: is it feasible? Disease markers 2018. 10.1155/2018/2082840 PMC6188592 30364165

[17] Moyer MW (2012) New biomarkers sought for improving sepsis management and care. Nature medicine 18: 999. doi: 10.1038/nm0712-999 22772541

[18] Alves-Filho JC, Spiller F, Cunha FQ (2010) Neutrophil paralysis in sepsis. Shock 34: 15–21. doi: 10.1097/SHK.0b013e3181e7e61b 20714263

[19] Cornbleet PJ (2002) Clinical utility of the band count. Clinics in laboratory medicine 22: 101–136. doi: 10.1016/s0272-2712(03)00069-6 11933571

[20] Ansari-Lari MA, Kickler TS, Borowitz MJ (2003) Immature granulocyte measurement using the Sysmex XE-2100: relationship to infection and sepsis. American journal of clinical pathology 120: 795–799. doi: 10.1309/LT30-BV9U-JJV9-CFHQ 14608908

[21] Kratz A, Maloum K, O’Malley C, Zini G, Rocco V, et al. (2006) Enumeration of nucleated red blood cells with the ADVIA 2120 Hematology System: an International Multicenter Clinical Trial. Laboratory hematology: official publication of the International Society for Laboratory Hematology 12: 63–70. 16751132

[22] Fang DZ, Sran G, Gessner D, Loftus PD, Folkins A, et al. (2014) Cost and turn-around time display decreases inpatient ordering of reference laboratory tests: a time series. BMJ quality & safety 23: 994–1000. doi: 10.1136/bmjqs-2014-003053 25165402

[23] Ardron MJ, Westengard JC, Dutcher TF (1994) Band neutrophil counts are unnecessary for the diagnosis of infection in patients with normal total leukocyte counts. American journal of clinical pathology 102: 646–649. doi: 10.1093/ajcp/102.5.646 7942630

[24] Lapić I, Padoan A, Bozzato D, Plebani M (2020) Erythrocyte sedimentation rate and C-reactive protein in acute inflammation: meta-analysis of diagnostic accuracy studies. American journal of clinical pathology 153: 14–29. doi: 10.1093/ajcp/aqz142 31598629

[25] Brigden ML (1999) Clinical utility of the erythrocyte sedimentation rate. American family physician 60: 1443–1450. 10524488

[26] Osei-Bimpong A, Meek J, Lewis S (2007) ESR or CRP? A comparison of their clinical utility. Hematology 12: 353–357. doi: 10.1080/10245330701340734 17654065

[27] Rast AC, Knobel D, Faessler L, Kutz A, Felder S, et al. (2015) Use of procalcitonin, C‐reactive protein and white blood cell count to distinguish between lower limb erysipelas and deep vein thrombosis in the emergency department: a prospective observational study. The journal of dermatology 42: 778–785. doi: 10.1111/1346-8138.12922 25982244

[28] Liu D, Su L, Han G, Yan P, Xie L (2015) Prognostic value of procalcitonin in adult patients with sepsis: a systematic review and meta-analysis. PloS one 10: e0129450. doi: 10.1371/journal.pone.0129450 26076027 PMC4468164

[29] Wacker C, Prkno A, Brunkhorst FM, Schlattmann P (2013) Procalcitonin as a diagnostic marker for sepsis: a systematic review and meta-analysis. The Lancet infectious diseases 13: 426–435. doi: 10.1016/S1473-3099(12)70323-7 23375419

[30] Póvoa P (2002) C-reactive protein: a valuable marker of sepsis. Intensive care medicine 28: 235–243. doi: 10.1007/s00134-002-1209-6 11904651

[31] Park BH, Kang Y, Park MS, Jung WJ, Lee SH, et al. (2011) Delta neutrophil index as an early marker of disease severity in critically ill patients with sepsis. BMC infectious diseases 11: 1–9. 22040292 10.1186/1471-2334-11-299 PMC3213213

[32] Lee H, Kim I-K, Ju MK (2017) Which patients with intestinal obstruction need surgery? The delta neutrophil index as an early predictive marker. Annals of Surgical Treatment and Research 93: 272–276. doi: 10.4174/astr.2017.93.5.272 29184881 PMC5694719

